# Conversion of failed internal fixation in proximal femur fractures using calcar-guided short-stem total hip arthroplasty

**DOI:** 10.1186/s10195-022-00655-6

**Published:** 2022-07-25

**Authors:** Yama Afghanyar, Marcel Coutandin, Michael Schneider, Philipp Drees, Karl Philipp Kutzner

**Affiliations:** 1 Department of Orthopaedics and Traumatology, St. Josefs Hospital Wiesbaden, Beethovenstr. 20, 65189 Wiesbaden, Germany; 2 Department of Orthopaedics and Traumatology, University Medical Centre of the Johannes Gutenberg University of Mainz, Langenbeckstraße 1, 55131 Mainz, Germany

**Keywords:** Conversion THA, Short stem, Femur fracture, Failed internal fixation, Optimys

## Abstract

**Purpose:**

Reoperations for secondary osteoarthritis, osteonecrosis, or hardware failure following failed internal fixation after intertrochanteric fracture (ITF) or femoral neck fracture (FNF) are common. An effective salvage treatment often involves complete removal of the hardware followed by total hip arthroplasty (THA). Almost no data are available regarding conversion to short-stem THA. This study aimed to evaluate clinical and radiological outcomes, potential complications, and the survival rate of short-stem THA following revision surgery.

**Methods:**

We investigated 27 patients who underwent conversion THA using a calcar-guided short stem. Patient-reported outcome measurements were obtained, including the Harris hip score, the Western Ontario and McMaster Universities Osteoarthritis Index, as well as pain and satisfaction on the visual analogue scale. Radiological follow-up was also performed.

**Results:**

We identified 18 (66.7%) patients diagnosed with FNF and 9 (33.3%) patients with ITF. Clinical and radiological outcomes were satisfactory at the last follow-up (30.56 ± 11.62 months). One patient required early revision surgery due to dislocation and greater trochanter fracture. At the last follow-up, none of the short stems required revision. No other major complications occurred.

**Conclusion:**

Given the low rate of complications and 100% survival, our findings indicate that short stems for conversion THA due to failed internal fixation may be considered an option in a properly selected patient population. However, it should not be considered a standard procedure and should only be performed by experienced surgeons.

## Introduction

Proximal femur fractures (PFFs) such as intertrochanteric fractures (ITFs) and femoral neck fractures (FNFs) are among the most frequent fractures in trauma surgery [1–3]. In young and middle-aged patients, internal fixation is the preferred treatment option for both injuries to preserve the native hip joint [4]. Although ITFs are most often treated with closed or open reduction and intramedullary fixation using proximal femoral nailing, FNF surgery in those patients usually involves a dynamic hip screw (DHS) or canulated screw fixation (SF) [4–6].

However, reoperations for secondary osteoarthritis, osteonecrosis, nonunion, and hardware failure are common [5–8]. In those cases, an effective salvage treatment for failed internal fixation often involves complete removal of the implanted hardware followed by total hip arthroplasty (THA), also known as conversion THA.

There is an ongoing debate on whether conversion THA should be considered a primary or revision THA [9]. Challenges include extensive surgical exposure, removal of the fixation material, potentially impaired bone quality, and secure fixation of the prosthetic implants.

Conversion after failed internal fixation most commonly employs conventional THA, either cementless or cemented [5, 10, 11]. Revision implants are needed in some cases [12]. For elderly patients, cemented conversion THA has been shown to offer advantages with regard to clinical outcomes and complications [11, 13]. However, the choice of THA is still controversial in younger patients.

Short-stem THA has gained popularity over the last decade and is now well established in most of Europe [14]. It focuses on metaphyseal anchorage and thus offers potential advantages regarding bone preservation and the prevention of stress shielding, and it provides favourable conditions for revision without altering the basic concepts of conventional THA [8, 15]. Encouraging results in recent years have broadened the range of indications for short-stem THA [16–18]. Although considered off-label use, the use of cementless short stems in assorted cases of revision surgery has been reported recently [15, 19, 20].

Almost no data are available regarding conversion to short-stem THA after failed internal fixation in PFF. The purpose of this study was to evaluate clinical and radiological outcomes, potential complications, and the survival rate of short-stem THA in revision surgery following failed internal fixation.

## Material and methods

**Fig. 1:**
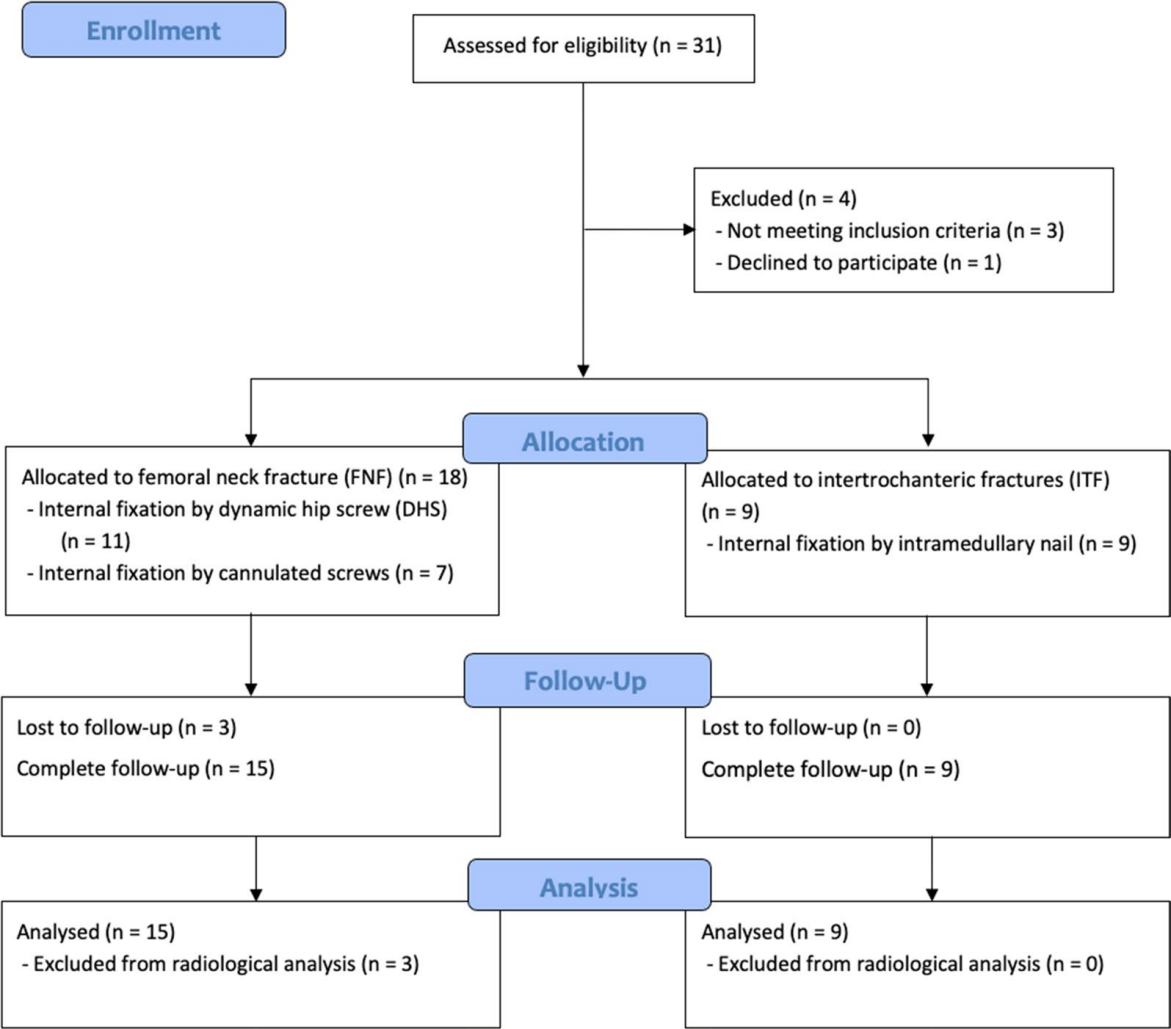
Flow chart of patient enrolment

This retrospective single-centre study included 27 patients following one-stage conversion THA using a calcar-guided short stem after failed internal fixation for PFFs between February 2016 and February 2020. A complete clinical and radiological follow-up was obtained at a minimum of 12 months for 18 patients. Six patients declined in-clinic follow-up examination due to the SARS-CoV-2 pandemic but were followed up by telephone. Three patients were lost to follow-up despite efforts to contact them. Thus, a total of 24 patients were investigated at the last follow-up (Fig. 1).

Ethical approval was obtained from the Institutional Review Board of the authors’ affiliated institution, and written consent to participate was obtained from all patients prior to enrolment.

### Surgical technique

**Fig. 2:**
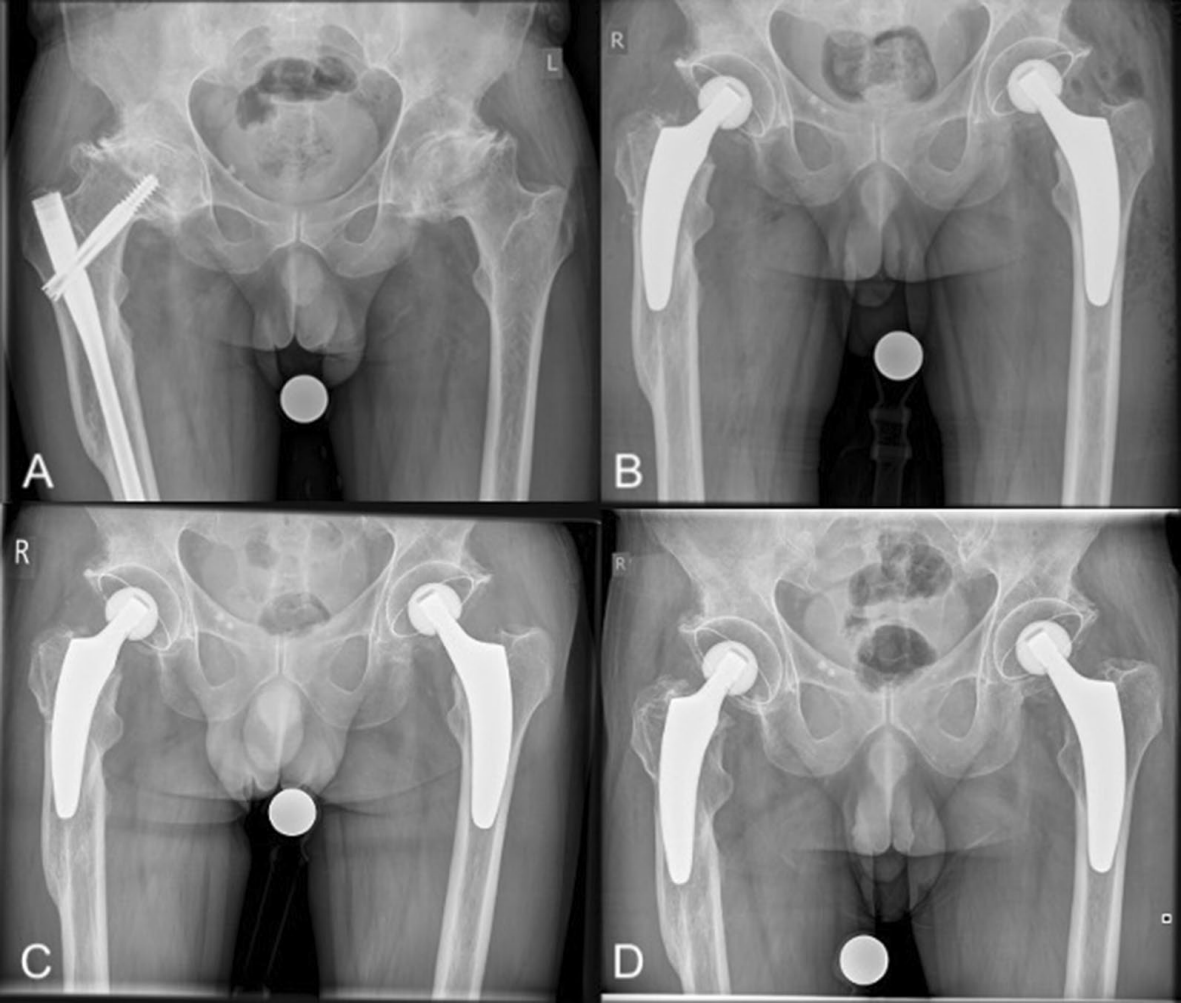
Radiographs of a 57-year-old male patient with secondary osteoarthritis (*right*) and primary osteoarthritis (*left*): **A** preoperative; **B** postoperative following one-stage bilateral THA; **C** 6-week follow-up; **D** 24-month follow-up (no signs of loosening)

**Fig. 3:**
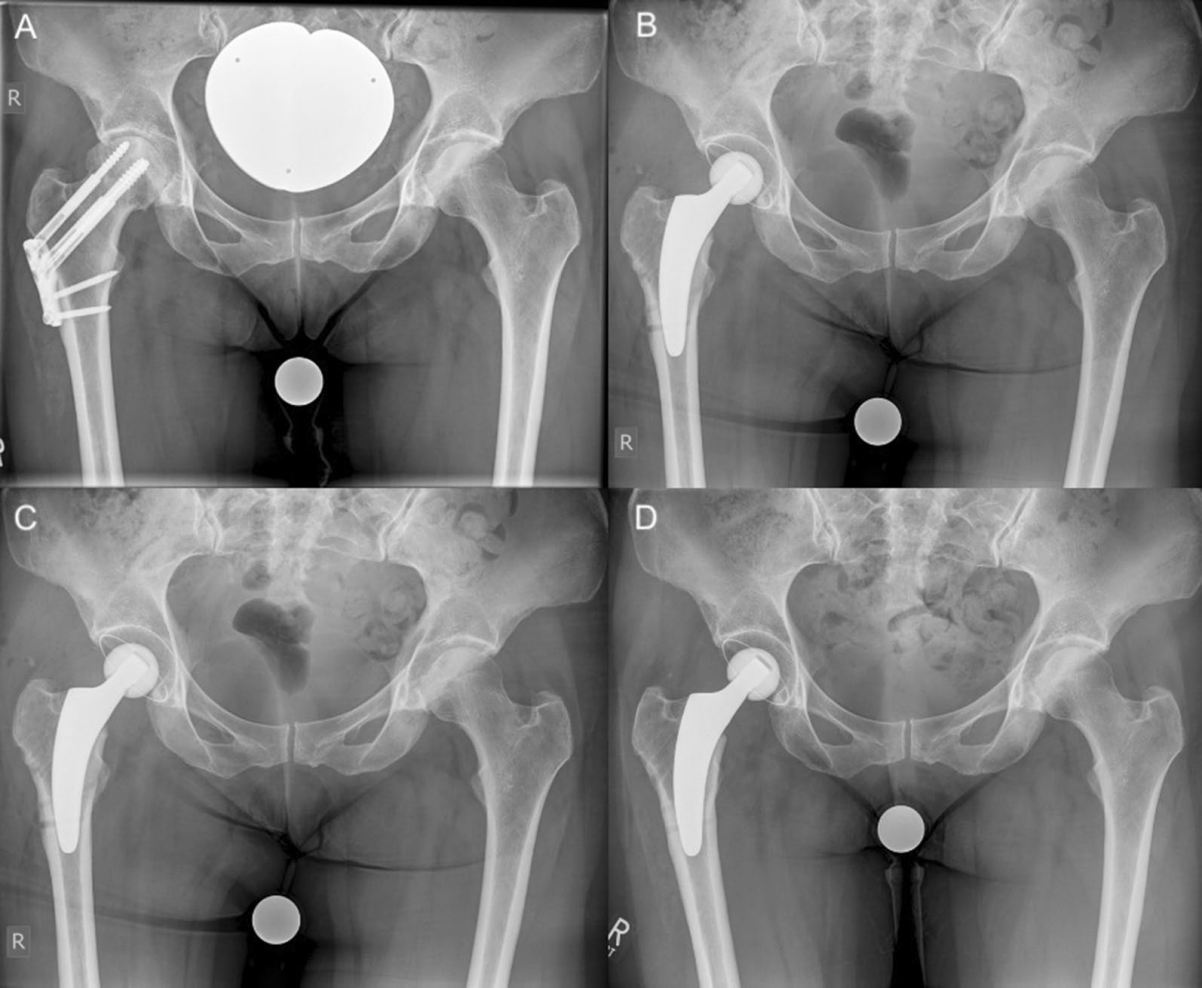
Radiographs of a 53-year-old female patient with secondary osteoarthritis: **A** preoperative; **B** postoperative; **C** 6-week follow-up **D**: 26-month follow-up (no signs of loosening or fracture)

**Fig. 4:**
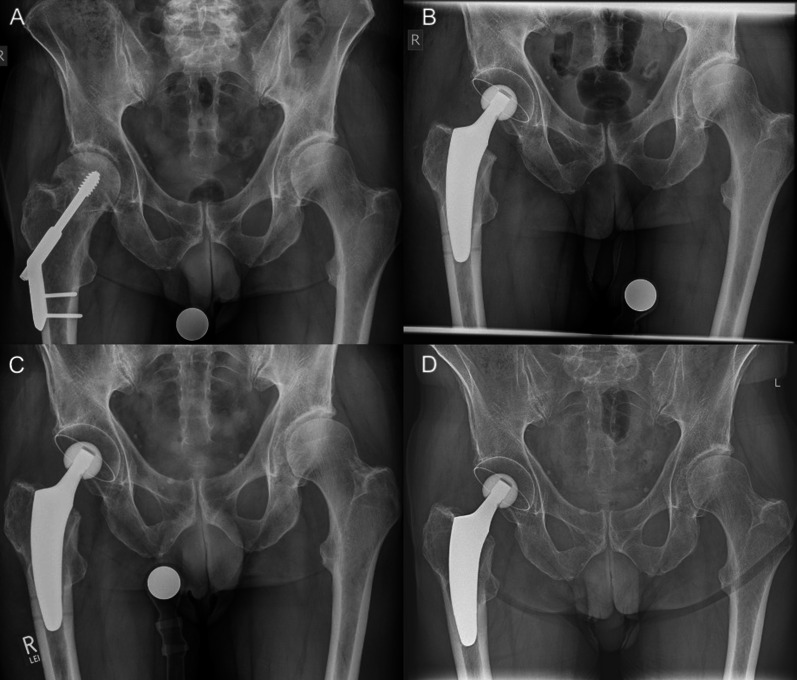
Radiographs of a 73-year-old male patient with hardware failure: **A** preoperative; **B** postoperative with fit-and-fill fixation; **C** 6-week follow-up; **D** 49-month follow-up (no signs of loosening or fracture)

All cases were revised using the anterolateral approach in the supine position, which is the standard approach at our institution; sometimes an additional incision distally for screw removal was needed. First, the complete clearance of previous hardware was accomplished. Afterwards, the osteotomy of the femoral neck was performed using an oscillating saw. This is a crucial step in order to determine the stem position [21]. Depending on the type of previous devices, the stem alignment was done individually to bypass screw holes and bone defects. In cases of intramedullary nail revision, the entry point of the blade was also bypassed, pursuing a metaphyseal anchorage to provide an individual load distribution (Figs. 2, 3). In cases involving DHS revision, a diaphyseal anchorage was pursued (Fig. 4). The biggest challenge was often the femoral preparation using implant-shaped rasps because of pre-existing sclerotic bone formations due to the previous implants. Reaming of the intramedullary canal was required in some of the cases. Assessment by intraoperative radiography is highly recommended to check the positioning of the rasp and avoid an incorrect stem position [22].

In all patients, one-stage conversion THA was performed using the calcar-guided short stem Optimys (Mathys Ltd., Bettlach, Switzerland). Selection criteria for the choice of the cementless short stem were primarily based on the femoral bone quality according to the Dorr classification [23]. Dorr types A and B were included, whereas Dorr type C was excluded. High age was not considered an exclusion criterion. Moreover, patients with a preoperatively diagnosed peri-implant infection followed by a two-stage procedure were excluded.

The short stem was either combined with a cementless primary monoblock cup (RM Pressfit Vitamys (*n* = 23); Mathys Ltd.) or a cementless primary metal-back cup (Fitmore (*n* = 4); Zimmer Biomet, Warsaw, IN, USA). Full weight-bearing was permitted postoperatively.

All patients completed a standardized preoperative screening regarding peri-implant infection, including a complete blood sample with C-reactive protein level and total leucocyte count. Preoperative hip punction and intraoperative tissue collection for microbiological and histological investigations were performed in all cases.

Standardized antero-posterior radiographs of the pelvis were obtained pre- and postoperatively as well as at regular follow-ups. Preoperative bone quality was classified according to the Dorr classification [23]. Postoperatively, the centrum–collum–diaphyseal (CCD) angle as well as the cup inclination was measured. Stem alignment was classified according to Kutzner et al. [24]. Further radiological follow-up was obtained for 18 patients.

Patient-reported outcome measurements (PROMs) were obtained at the last follow-up, including the Harris hip score (HHS; range from 0 to 100, with ≥ 90 = excellent and < 70 = poor), the Western Ontario and McMaster Universities Osteoarthritis Index (WOMAC; range from 0% = best to 100% = worst), as well as pain (0 = no pain to 10 = worst pain possible) and satisfaction (0 = worst to 10 = best) on the visual analogue scale (VAS). The EQ-5D-5L (EuroQol Group) was used to assess health status. Patients who declined in-clinic follow-up completed all questionnaires except the HHS.

Statistical analysis was carried out with SPSS version 26 (IBM, Armonk, NY, USA). Continuous variables are expressed as the mean ± standard deviation (SD) and range. Additional data are reported as the frequency and percentage. Pre- and postoperative differences in HHS were examined nonparametrically using Wilcoxon signed-rank tests. Differences were considered statistically significant at *p* < 0.05.

## Results

We identified 18 (66.7%) patients with FNF and 9 (33.3%) patients with ITF. Internal fixation in all patients with ITF was performed by intramedullary nailing. In the FNF group, 11 (40.7%) patients were treated with a DHS and 7 (25.9%) patients with cannulated screw fixation. Causes of failure were secondary osteoarthritis in 17 (63%) cases, osteonecrosis in 7 (25.9%) cases, and hardware failure in 3 (11.1%) cases.

The mean follow-up duration was 30.56 ± 11.62 months (range 12–49 months). The mean age at revision surgery was 65.26 ± 10.34 years (range 49–80 years). Further demographic details are presented in Table 1. Overall, 22 (81.5%) patients were classified as Dorr type A, and 5 (18.5%) patients were classified as Dorr type B.

**Table 1:** Demographic and clinical data

Demographics (*n* = 27)	Value [mean ± standard deviation (minimum–maximum) or *n* (%)]
Age at revision	65.3 ± 10.3 years (49–80 years)
Gender	14 (52%) male; 13 (48%) female
ASA grade	
1	8 (29.6%)
2	14 (51.9%)
3	5 (18.5%)
BMI	25.6 ± 3.8 kg/m^2^ (19.4–35.8 kg/m^2^)
Duration of follow-up	30.6 ± 11.6 months (12–49 months)
Fracture diagnosis	
Femoral neck fracture	18 (66.7%)
Intertrochanteric fracture	9 (33.3%)
Type of osteosynthesis	
Dynamic hip screw	11 (40.7%)
Cannulated screw fixation	7 (25.9%)
Proximal femoral nail	6 (22.2%)
Gamma nail	3 (11.1%)
Revision diagnosis	
Secondary osteoarthritis	17 (63%)
Osteonecrosis of the femoral head	7 (25.9%)
Hardware failure	3 (11.1%)
Haemoglobin value	
Preoperative	14.3 ± 1.1 g/dl (11.6–16.1 g/dl)
Postoperative	10.8 ± 1.2 g/dl (8.2–13.6 g/dl)

*ASA* American Society of Anesthesiologists, *BMI* body mass index

The mean haemoglobin value decreased from 14.3 ± 1.1 mg/dl (range 11.6–16.1 mg/dl) before surgery to 10.8 ± 1.2 mg/dl (range 8.2–13.6 mg/dl) postoperatively. None of the patients required a blood transfusion.

### Clinical evaluation

The mean HHS at baseline was 42.56 ± 6.37 (range 30–59) and improved significantly to 96.78 ± 5.01 (range 79–100) at the last follow-up (p < 0.001). The outcomes of 16 patients were excellent (HHS ≥ 90), and those for 2 patients were only moderate (HHS 71 and 79). All PROMs and clinical outcomes are summarized in Table 2.

**Table 2:** Functional scores at last follow-up

	HHS (%)	WOMAC (Index)	EQ-5D-5L	Pain (VAS)	Satisfaction (VAS)
Mean	96.78	2.17	0.98	0.3	9.5
Standard deviation	5.01	5.13	0.06	0.8	0.9
Minimum	79	0	0.72	0	6
Maximum	100	24	1.00	3	10

*EQ-5D-5L*, EuroQol Group, *HHS* Harris hip score, *VAS* visual analogue scale, *WOMAC* Western Ontario and McMaster Universities Osteoarthritis Index

### Radiological evaluation

In most cases, a valgus or neutral stem alignment was observed, given that over 90% of the hips were classified group C, D, or E according to Kutzner et al. [24] (Table 3). The mean cup inclination was 44.3° ± 3.5 (range 39–51°). At the last follow-up, there were no radiological signs of subsidence, aseptic loosening, radiolucent lines, stress shielding, or fracture (Figs. 2, 3, 4).

**Table 3:** Postoperative stem alignment according to the classification of Kutzner et al. [24]

CCD category	*n*	%
A (< 124.9°)	0	0.0
B (125–129.9°)	2	7.4
C (130–134.9°)	5	18.5
D (135–139.9°)	6	22.2
E (> 140°)	14	51.6
Total	27	100

### Complications

**Fig. 5:**
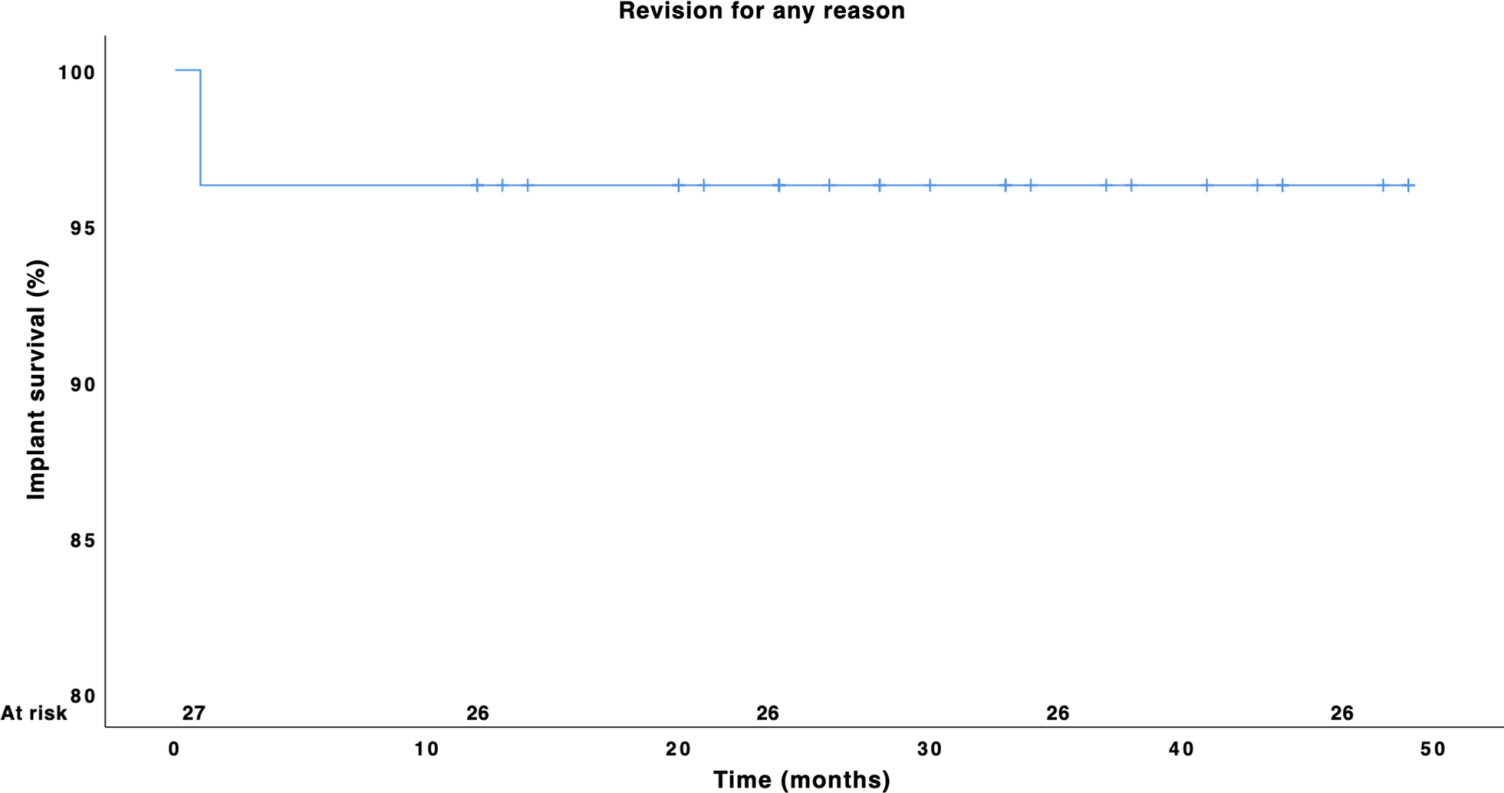
Kaplan–Meier survival plot for the endpoint of revision for any reason

One patient required early revision surgery due to dislocation and greater trochanter fracture, which included the implantation of a cemented dual mobility acetabular component (Avantage, Zimmer Biomet) and a cerclage of the greater trochanter. No further revision surgery was necessary. One patient showed persistent femoral nerve palsy during follow-up, but no further major complications occurred. At the last follow-up, none of the short stems required revision, corresponding to 100% stem survival. Overall, the survival rate for the endpoint of revision for any reason at last follow-up was 96.3%. The Kaplan–Meier survival plot is shown in Fig. 5.

## Discussion

The purpose of this study was to highlight our experience using short-stem THA following failed internal fixation for PFF and to investigate potential risks and complications. No previous study has reported on one-stage short-stem THA in revision surgery after osteosynthetic hardware removal. PROMs and radiological results at the last follow-up were encouraging, along with high satisfaction rates. One patient with early dislocation and fracture of the greater trochanter required a change of the acetabular component and refixation of the greater trochanter. No other revision surgery was needed. Stem survival was 100% at the last follow-up.

Conversion THA is considered a salvage procedure with technical challenges and perioperative complications. Previous attempts were made to classify conversion THA as primary or revision THA. On the one hand, it has been suggested that conversion THA should be considered primary THA because, although it is a technically challenging procedure, it yields fewer orthopaedic complications compared to revision THA [25]. For example, in 2020, a matched cohort study by Vles et al. found that most (72%) of the patients who underwent conversion THA were treated with primary implants and never experienced a major complication [26].

On the other hand, it requires the removal of internal fixation devices and previous implants [9, 27]. Thus, conversion has often been associated with increased operative time and blood loss, increased fracture, dislocation, and infection rates, and lower functional outcome scores compared to primary THA [9]. Potential postoperative complications may therefore negatively influence the revision rate of conversion THA. Gjertsen et al. [28] analysed data from the Norwegian Arthroplasty Registry and found a higher revision rate for THA performed following failed internal fixation compared with acute fracture THA or primary THA. Dislocation and periprosthetic fracture were the most common causes of revision. Leonardsson et al. [6] examined data from the Swedish Hip Arthroplasty Registry and also found a high revision rate when comparing acute THA in fracture and THA performed after failed internal fixation (2.9% vs 4.4%). The most common causes of re-revision were dislocation and periprosthetic fracture, and the type of femoral component and surgical approach influenced the revision risk [6].

To date, in almost all data on conversion THA, either conventional straight stems or revision stems, cementless as well as cemented, were used. As far back as 2004, Zhang et al. reported on 19 patients who underwent conversion THA for failed internal fixation of intertrochanteric fractures [29]. Complications were common, given seven cases of intraoperative fracture of the greater trochanter and three cases of postoperative dislocation. HHS was 79.8 at the latest follow-up. Archibeck et al. published a series of 50 cases following failed internal fixation of proximal femoral fractures with a minimum 2-year follow-up in 2013 [5]. They found 12 patients who had early surgical complications related to the procedure, such as dislocations and periprosthetic fractures. At last follow-up, mean HHS was 81.8.

Over the last decade, short-stem THA has gained popularity in Germany, as well as in large parts of Europe, and is already used in > 10% of all primary cases [30]. Potential advantages compared to conventional THA are bone preservation and soft-tissue sparing [21]. Faster postoperative mobilization and a reduced hospital stay have been reported, as well as less blood loss and lower transfusion rates [31, 32]. Concurrently, indications for short-stem THA have constantly been expanded in recent years [14]. Although the use of short stems in revision surgery is considered off-label use, they may be selected to reduce surgical trauma and preserve as much femoral bone stock as possible.

Coutandin et al. recently introduced the concept of downsizing the femoral component in revision THA in a subset of cases using the same short stem as in the present study, and reported satisfying clinical outcomes and no major complications [15]. Another case series investigated the outcomes of revision surgery of failed hip resurfacing arthroplasty using short-stem THA. The authors concluded that cementless short stems may be considered as a revision implant for experienced surgeons [20]. Furthermore, Bostian et al. [19] published a surgical technique that uses short stems in cases of challenging proximal femoral anatomy, excessive femoral bowing, diaphyseal deformities, and retained implants.

The present case series supports the use of the investigated stem design in conversion THA. Only one case needed re-revision due to dislocation and greater trochanter fracture. None of the short stems had to be revised at the last follow-up. The design of the investigated short stem may account for potential advantages compared with earlier short-stem designs, particularly in cases with reduced bone quality. In addition to the metaphyseal fixation, this stem design enables the surgeon to intentionally choose an additional fit-and-fill in the proximal diaphysis [14] (Fig. 4) by applying a neutral or valgus alignment instead of a varus positioning. This may lead to increased safety for certain indications, such as conversion THA [33]. Moreover, operation time and blood loss could be held at low levels with short stems, and there was no need for blood transfusions, which corresponds to the current literature [32]. Given an HHS of 96.8, the clinical outcomes, including PROMs, were found to be superior compared to the available literature [5, 29].

The results of the present investigation are supported by the only previous study with data on the usage of short-stem THA in conversion THA. De Meo et al. reported conversion THA outcomes after failed fixation of intracapsular compared to extracapsular hip fractures and included seven cases in which a short stem was used [34]. No complications were observed during follow-up. They concluded that the physiological load distribution in the metaphyseal bone may reduce stresses in the cortical region, where the screw holes could create minor resistance points [34].

Although one of the major concerns when using a proximal anchoring short stem in conversion THA following internal fixation is the imminent risk of periprosthetic fracture in the region of prior screw holes, remarkably, there were no intraoperative or early postoperative fractures in the present series.. Archibeck et al. [5] recommended prophylactic cable placement in this region to avoid this issue. Haidukewych and Berry [7] advised bridging the cortical screw holes with long-stemmed femoral implants. Similarly, Winemaker et al. [35] recommended diaphyseal anchorage of the stem. In a biomechanical analysis performed by Chen et al. [36], a bypass of the last screw hole by at least 3 cm was recommended.

However, the superiority of the above-mentioned technical modifications was not substantiated by the results of the current study.

Another crucial step in conversion THA is excluding peri-implant infection as a cause of failed internal fixation. Besides preoperative blood work-up, hip punction is recommended to determine the need for a one- or two-stage conversion THA strategy [37]. Hemmann et al. [38] recently evaluated the infection risk for conversion THA by a one-stage procedure in the absence of clear infection signs. A positive microbiological test result was found in 10% of the cases of one-stage conversion THA, and a postoperative periprosthetic infection rate of 5.8% was reported. The present study only included patients without preoperative proof of a peri-implant infection who qualified for a one-stage procedure, as intraoperative microbiological testing using swabs or sonication was not used routinely at that time. However, no signs of periprosthetic infection were obvious in any patients at the last follow-up, supporting the one-stage strategy in those cases.

The present study has several limitations. First, the retrospective study design and small series are inherent limitations. However, there are almost no data available on the use of short stems in conversion THA, making the present results interesting for the orthopaedic community, even if only a small cohort is represented. Second, the lack of a control group limits a direct comparison to conventional conversion THA. Third, the short-term follow-up does not allow definite conclusions to be drawn about the safety of short-stem THA in revision surgery. Further studies with long-term data and larger populations are needed to confirm these findings. Finally, the current investigation only reflects the results of one stem design.

## Conclusion

Given the low rate of complications and 100% survival at the last follow-up, our findings indicate that short-stem THA for conversion THA due to failed internal fixation may be considered an option in a properly selected patient population. However, conversion THA using a short stem should not be considered a standard procedure and should only be performed by experienced surgeons. Caution should be used in drawing final conclusions based on the present results, as the follow-up was relatively short and long-term results are lacking.

## Declarations

### English editing service

The manuscript was edited for correct English language usage by qualified native English-speaking editors at Charlesworth Author Services.
